# Pathophysiology of Lysosomes in a Nutshell

**DOI:** 10.3390/ijms241310688

**Published:** 2023-06-26

**Authors:** Ciro Isidoro

**Affiliations:** Laboratory of Molecular Pathology and NanoBioImaging, Department of Health Sciences, Università del Piemonte Orientale, 28100 Novara, Italy; ciro.isidoro@med.uniupo.it

Lysosomes are acidic organelles present in all nucleated mammalian cells. They represent the final destination for the degradation of extracellular and intracellular material therein delivered via the endocytic and autophagy pathways, respectively [1].

The endosomal-lysosomal system also includes the early endosomes, that can recycle back to the cell surface the membrane-bound receptors after having released the endocytosed ligand, and the late endosomes, also known as multivesicular bodies because they can contain small vesicles that are eventually secreted as exosomes [2]. Exosomes serve an important function in local and distant cell-to-cell communication since they bring partially processed molecules with signaling properties [2].

Lysosome-related organelles with secretory functions are present in highly differentiated immune cells such as the lymphocytes and mast cells, and this highlights the importance of lysosome biogenesis for an efficient immune response [3,4].

The lysosome was at the beginning considered the ‘waste bin’ for the degradation of foreign material. It was also considered a ‘suicide bag’ that would lead to necrotic cell death in case of rupture. Later, it has been recognized that lysosomes play a crucial role in macromolecular and organelle turnover and, by extension, in cell and tissue homeostasis [5]. In fact, thanks to the wide range of acidic hydrolases almost all the biomolecules and bioparticles delivered to it via autophagy or endocytosis/phagocytosis can be degraded [6,7]. Accordingly, the genetic lack of a lysosomal enzyme would result in the abnormal accumulation of undigested substrate and a consequent lack of downstream products. This characteristically leads to a lysosomal storage disease that compromises the physiological development and differentiation of organs and tissues [8].

Inadequate digestion and processing and/or abnormal proteolysis of substrates due to insufficient expression of a lysosomal enzyme, insufficient acidification, or altered trafficking within the endosomal–lysosomal compartments inevitably impact cell functioning [9,10,11]. On the other hand, abnormal secretion of lysosomal enzymes causes an excessive degradation of the extracellular matrix, which compromises the architecture and functioning of the tissues [12,13].

From the above, it appears clear that defective biogenesis or malfunctioning of this organelle would negatively impact general homeostasis and health. Thus, understanding the biology and functional role of lysosomes can be leveraged for the therapeutic benefit of lysosome-related diseases.

This Special Issue collects fourteen articles highlighting the importance and role of the endosomal–lysosomal compartments and their connections with the autophagy, endocytosis, and exocytosis pathways in various pathophysiological aspects. Below is a summary of the topics covered. 

Durso et al. [14] investigated the changes in lysosome dimension and dynamics during neuronal stem cell (NSC) differentiation. They found that during NSC differentiation, the average diameter of the lysosomes halved and that their subcellular localization shifted from the soma to newborn projections.

In their study, Villamil Giraldo et al. [15] employed a pH-sensitive lysosomotropic detergent to obtain insights into the mechanisms of lysosomal membrane permeabilization that may lead to cell death. 

The work by Canonico et al. [16] shows that rapamycin, an inhibitor of mTOR and, thereby, activator of autophagy, can inhibit the toxin of the Gram-negative *Campylobacter jejuni*, which affect lysosome positioning and function, thus avoiding sub-lethal effects on macrophage-like cells. 

In their review, Buratta et al. [17] focus on the crosstalk between the autophagy and the endosomal–lysosomal pathways and point to the pathophysiological relevance of the intersection between degradative and secretory functions leading to the release of exosomes.

Baht et al. [18] focused on the role of mucolipin-1 in lysosome positioning and secretion of exosomes in aortic smooth muscle cells. In a model of arterial medial calcification, in which there is an abnormal release of exosomes from vascular smooth muscle cells, they found a reduced interaction of lysosome and multivesicular bodies along with an increased secretion of exosomes when mucolipin-1 was knocked out. 

To determine the role of autophagy in protecting the proximal tubular cells from acute renal injury, Suzuki et al. [19] generated transgenic knockout mice for ATG7, which is necessary for autophagosome formation, specifically in renal proximal tubular cells. They found that with aging, the mice developed renal injury where the tubular cells accumulated inclusions positive for p62, a marker of the autophagy cargo. 

In a separate study, Suzuki et al. [20] investigated the protective role of lysosomal cathepsin D in acute kidney injury. To this end, they employed a conditional knockout *CtsD^flox/−^*; *Spink3^Cre^* mouse where cathepsin D was deficient in its renal proximal tubular cells. In these cells, the autophagy degradation was impaired with the accumulation of autophagosomes and inclusions, increasing their sensitivity to ischemia/reperfusion injury. 

Wong [21] presents a comprehensive review of the essential role of endosomal–lysosomal proteolysis in astrocytes, particularly of proteins associated with Alzheimer’s disease (amyloid precursor proteins, Apolipoprotein E, and tau), Parkinson’s disease (alpha synuclein), and Huntington disease (huntingtin). 

To study the role of lysosomal proteolysis, particularly when mediated by cathepsin B, in the formation of synapses and cognitive abilities, Hwang et al. [22] employed transgenic APP/PS1 (amyloid precursor protein/presenilin-1) mice, a model resembling familial Alzheimer’s disease, and other mouse models resembling Parkinson’s disease and mild cognitive impairment. They found that stimulation of cathepsin B-mediated clearance of amyloidogenic peptides and α-synuclein in the affected neurons could ameliorate the compromised synapses and counteract the cognitive decline in the animals.

Pompe disease is a lysosomal storage disease where glycogen accumulates within the lysosomes due to the genetic lack of acid maltase or acid α-glucosidase. Fusco et al. [23] reviewed the current knowledge on the respiratory phenotype of Pompe disease in the mouse model.

The functional and structural integrity of podocytes is essential for glomerular filtration and urine formation, and the autophagy–lysosomal system plays an important role in podocyte homeostasis. Li et al. [24] reviewed the current knowledge about how dysregulation of lysosomal function in podocytes underlies chronic glomerular diseases.

Chi et al. [25] present a review highlighting the role of the autophagy–lysosomal pathway in cardiomyocytes and how its dysregulation leads to cardiovascular diseases. 

Obesity is associated with inflamed adipose tissue and the release of adipokines and cytokines, which eventually alter the metabolism in distant organs, thereby facilitating the onset of type 2 diabetes, non-alcoholic fatty liver disease, and cardio-and cerebrovascular diseases, ultimately developing a metabolic syndrome. In their review, Mizunoe et al. [26] highlight the abnormality of the lysosomal degradative functions in white adipose tissue and the liver that occur in obesity, with insights on possible strategies for modulating the lysosomal function to prevent metabolic syndromes.

Rudzińska et al. [27] review the current literature supporting the involvement of lysosomal cathepsins B and L in cancer progression, angiogenesis, metastasis, and chemoresistance.
